# Knowledge, attitudes and practices on schistosomiasis and soil-transmitted helminths among caregivers in Ingwavuma area in uMkhanyakude district, South Africa

**DOI:** 10.1186/s12879-019-4253-3

**Published:** 2019-08-22

**Authors:** Hlengiwe Sacolo-Gwebu, Muhubiri Kabuyaya, Moses Chimbari

**Affiliations:** School of Nursing and Public Health, College of Health Sciences, Howard College Campus, University of KwaZulu-Natal, Durban, South Africa

**Keywords:** Knowledge, Attitudes, Practices, Schistosomiasis, Soil-transmitted helminths, Caregivers, Community members, uMkhanyakude district, South Africa

## Abstract

**Background:**

Schistosomiasis and soil-transmitted helminth infections are among the most chronic infections worldwide. Based on their demonstrable impact on human health, the WHO recently recommended the implementation of robust strategies aimed at controlling or eliminating schistosomiasis and soil-transmitted helminths by 2020. The implementation of this strategy, however, warrants a clear understanding of the community’s knowledge, attitudes and practices in relation to these infections. This study sought to identify sociocultural gaps that should be addressed to ensure the success of cost-effective community-based schistosomiasis-soil-transmitted helminths control and elimination programs.

**Methods:**

This was a cross-sectional mixed methodology study. Quantitative data were collected using a structured questionnaire from 442 caregivers of preschool aged children. In-depth interviews and focus group discussions were conducted among caregivers, preschool teachers, traditional authorities and community caregivers. All interviews were captured using an audio recorder to maximize accuracy. Quantitative data were analysed using bivariate and multivariate techniques while qualitative data were analysed thematically.

**Results:**

Findings reflected inadequate knowledge, attitudes and practices in relation to schistosomiasis and soil-transmitted helminths while awareness of schistosomiasis and soil-transmitted helminths was high (87.1 and 79.2% respectively). Correct knowledge on transmission, prevention, signs and symptoms and life cycle was low (below 50%) for both infections among those who had heard of the disease. From multivariate analysis, being aged at least 35 years increased the odds of reporting good practices on schistosomiasis by 65% (COR 1.652, 95% CI: 1.073–2.543) while receiving health information through community meetings (COR 0.072, 95% CI: 0.010–0.548) significantly reduced the odds of having good knowledge on schistosomiasis.

**Conclusions:**

These findings are valuable in designing behavioural change approaches towards enhancing health outcomes through community-based interventions to ensure effective control and elimination of schistosomiasis and soil-transmitted helminths. There is a critical need for channelling efforts towards making health education the core of schistosomiasis and soil-transmitted helminths programs aimed at achieving intensified control or elimination of these infections by 2020.

**Electronic supplementary material:**

The online version of this article (10.1186/s12879-019-4253-3) contains supplementary material, which is available to authorized users.

## Background

Schistosomiasis and soil-transmitted helminths (STH) are the most prevalent neglected tropical diseases (NTD) among disadvantaged communities in rural areas [1]. Sub-Saharan Africa (SSA) carries the heaviest burden of schistosomiasis and STH estimated at 90% of the global prevalence [2]. Schistosomiasis affects at least 200 million people [3] whilst STH affects more than a billion people globally [4]. Although schistosomiasis and STHs share significant similarities [5, 6], their life cycles and treatment strategies differ. For instance, schistosomiasis requires an intermediate host snail to complete its cycle and praziquantel for treatment whilst STHs complete their life cycle in the soil and Albendazole for treatment. Schistosomiasis and STH are associated with negative acute and chronic physical and cognitive outcomes. The long-term impact of these infections may cause permanent disability or even death in some cases.

Schistosomiasis and STH can be controlled using three key approaches which include improved sanitation, mass drug administration and health education [7, 8]. Other interventions for infection prevention include access to safe water, basic sanitation, and the promotion of hygiene practices [9–12]. These interventions play a critical role in the prevention and successful control or elimination of schistosomiasis and STH. Due to the commonalities between schistosomiasis and STH, their modes of prevention and transmission are often confused by community members and school aged children (SAC). The integration of schistosomiasis and STH control programs is therefore important to ensure that clear health education messages are crafted and disseminated. The success of community-based control initiatives depends on community acceptance of the programme [10, 13]. This implies that programs should first seek to understand the community’s knowledge, attitudes and practices (KAP) towards schistosomiasis and STH before implementing program activities. Among community members, caregivers (parents and guardians) are considered a critical group due to their functional role as caregivers. Caregivers are highly influential within their families and at the community level, however without enough knowledge, they tend to mislead others or engage in risky practices that predispose their young children to schistosomiasis and STH infections [14, 15].

In rural areas of KwaZulu-Natal in South Africa, where schistosomiasis [16, 17] and STHs [18] are endemic, community-based KAP studies on schistosomiasis and STH are limited. This negatively impacts the effectiveness and sustainability of the control interventions aiming at eliminating schistosomiasis and STH at the community level [19]. According to recent findings, caregivers are critical in the control of schistosomiasis and STH among preschool aged children (PSAC) [15]. Studies conducted in Nigeria reported that caregivers predispose their children to schistosomiasis through risky water-related practices [20]. There is however lack of detailed understanding of the underlying reasons for such behaviour among caregivers. A study that reviewed KAP on schistosomiasis in sub-Saharan Africa (SSA) indicated the importance of including caregivers in the planning and implementation of control programmes for enhanced efficiency among PSAC [15]. The aim of this study, therefore, was to investigate the knowledge, attitudes and practices on schistosomiasis and soil-transmitted diseases among community members of the Ingwavuma area in uMkhanyakude District, South Africa.

## Methods

### Study area

The Ingwavuma area is in the North-Western part of uMkhanyakude District in KwaZulu-Natal, South Africa. It is arid and sub-tropical marked by erratic rainy and dry seasons. It also characterised by a hot and humid summer from November to February and a cooler, drier winter from June to August [21]. The hydrological network is formed by the Pongola and Ingwavuma rivers. Most communities in the Ingwavuma have limited access to piped water and as such depend on rivers, dams and ponds to sustain their domestic water needs [22].

### Study design and sampling

#### Questionnaire survey

**Fig. 1:**
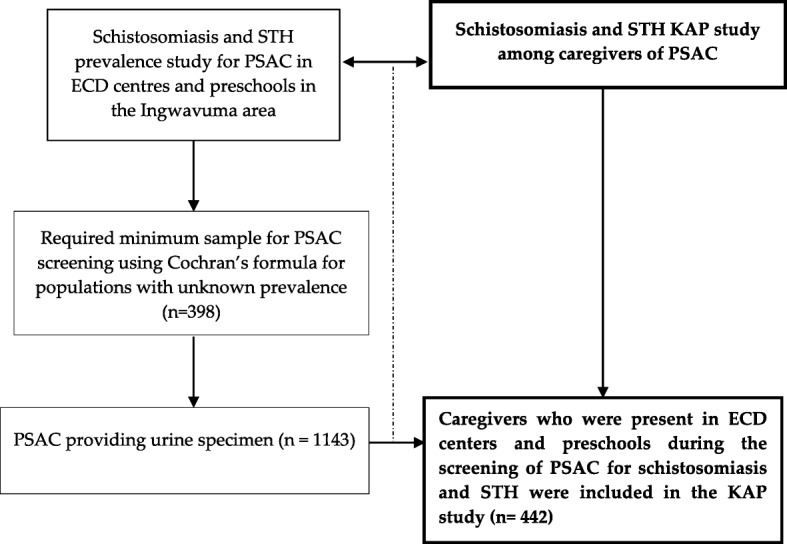
KAP survey sampling framework

We conducted a cross-sectional study among caregivers who were present in preschools and ECD centres during the screening of their PSAC under their care for schistosomiasis and STH. Participants belonged to villages located in the north-western part of that Ingwavuma area that was purposely selected into the study based on previous reports of schistosomiasis [22] and STH [23] among SAC in the area. The sample size for this study was linked to the sample size determined for the PSAC screening through the Cochran formula [24] for populations with unknown disease prevalence to yield a representative sample for larger populations. The minimum sample size required size through Cochran’s formula calculation was 398. However, all caregivers present on the day of PSAC screening in ECD centres and preschools were recruited into the study (Fig. 1).

#### Key informant in-depth interviews

In-depth interviews were conducted among 62 key informants (traditional authorities, preschool teachers, ECD practitioners, community caregivers (CCGs) and nurses within the Ingwavuma area. The KAP key informant interview guide was developed, pre-tested, amended accordingly and used to interview the selected participants on various KAP aspects relating to schistosomiasis and STH.

#### Focus group discussions

There were 138 participants who participated in 11 FGDs, averaging to at least 10 participants per FGD. The size of FGDs was within the range of the recommended size of 8 to 12 participants for FGDs. We conducted focus group discussions (FGDs) categorized by gender among caregivers of PSAC (8 FGDs) and CCGs (3 FGDs). The FGDs were conducted and led by trained moderators and note-takers fluent in isiZulu, the local language. Participants were purposively selected based on similar characteristics. Data collected were analysed thematically. Emerging themes and sub-themes were used to probe community members, whilst those taking notes focused on capturing non-verbal information and expressions that were evident. At the end of each interview, the research team held a short meeting to validate the collected information. FGDs were conducted in four main villages, purposely picked due to prior reported high prevalence among school aged children within our study area [22, 25, 26]. In each selected village, two FGD discussions were conducted comprising of ten members each as recommended by previous authors [27]. FGDs were grouped by gender nature of a rural setting where females are expected to be reserved in the presence of men.

### Data collection and management

Quantitative data was collected using KoBo Collect software [28] and subsequently imported into SPSS. Statistical analysis was done after data validation. Qualitative data from interviews was transcribed per verbatim and typed into an excel spreadsheet. A code sheet was developed for the focus group discussions after which, the data was coded into selected themes and was analyzed. Researchers checked for consistency of the transcripts against the audio files to ensure the accuracy of the transcribed files.

#### Outcome variables

The study assessed knowledge, attitudes and practices on STH and schistosomiasis. Firstly, total scores were determined by aggregating scores from responses to questions on knowledge and practices. Average scores were then calculated by dividing the total score by the number of questions in the respective attribute. Knowledge was defined as low or high level for scores below and above average. Similarly, practices were defined as poor or good practices for scores below and above the average score. We conducted a reliability test on the tool that assessed attitudes on schistosomiasis and STH comprising of14 items. Using Cronbach’s alpha, the tool achieved a reliability score of α = 0.704 and was deemed reliable. All items appeared to decrease the alpha when deleted from the selection and were, therefore, retained for the study.

#### Data analysis

Chi-square test was used to assess associations between variables. A *p*-value of < 0.05 was considered statistically significant. Multivariate analysis was conducted to assess the effect of background characteristics on practices and knowledge. Significant variables from the chi-square test were used as predictors in the multivariate analysis on practices and knowledge levels for both schistosomiasis and STH. Results from the binary logistic regression were expressed as crude odds ratios (COR) and adjusted odds ratio (AOR) with their 95% confidence intervals (CI). The qualitative data were analyzed using content and thematic analysis to identify emerging themes. The process of analysis involved acquaintance with the data and development of codes based on our research findings.

## Results

### Sociodemographic characteristics of PSAC caregivers

Table 1 presents the overall sample characteristics. A total of 442 caregivers comprising of 28 (6.3%) males and 414 (93.7%) females participated in the study. The participants were caregivers to 442 PSAC that had been screened for schistosomiasis and STH. The majority (63.1%) of the caregivers were in the 15–34 year age group while 364 (82.4%) of the caregivers were unmarried.

**Table 1:** Caregivers’ knowledge of schistosomiasis and STH and their main source of information

Caregivers’ knowledge of schistosomiasis and STH	Caregivers main source of information on schistosomiasis and STH						
	None	Clinic	Research organization	Community meeting	Media	Relatives/friends	*X* ^*2*^ *p-value*
Schistosomiasis transmission							
No idea	51 (89.5)	27 (58.7)	38 (53,5)	35 (38.5)	33 (58.9)	60 (49.6)	0.001
Incorrect answers	0 (0.0)	3 (6.5)	11 (15.5)	15 (16.5)	7 (12.5)	27 (22.3)	
At least one correct answer	6 (10.5)	16 (34.8)	22 (31.0)	41 (45.1)	16 (28.6)	34 (28.1)	
Schistosomiasis prevention							
No idea	49 (86.0)	7 (37.0)	26 (36.6)	33 (36.3)	8 (32.1)	49 (40.5)	0.001
Incorrect answers	1 (1.8)	4 (8.5)	8 (11.3)	10 (11.0)	15 (26.8)	21 (17.4)	
At least one correct answer	7 (12.3)	25 (54.3)	37 (52.1)	48 (52.7)	23 (42.1)	51 (42.1)	
Schistosomiasis signs and symptoms							
No idea	57 (100.0)	12 (26.1)	24 (33.8)	31 (43.1	5 (26.8)	37 (30.6)	0.001
Incorrect answers	0 (0.0)	1 (2.2)	3 (4.2)	22 (24.2)	9 (16.1)	27 (22.3)	
At least one correct answer	0 (0.0)	33 (71.7)	44 (62.0)	38 (41.8)	32 (57.1)	57 (57,1)	
STH transmission							
No idea	28 (49.1)	27 (58.7)	43 (60.6)	38 (41.8)	31 (55.4)	74 (61.2)	0.157
Incorrect answers	9 (15.8)	5 (10.9)	11 (15.5)	16 (17.6)	11 (19.6)	21 (17.4)	
At least one correct answer	20 (35.1)	14 (30.4)	17 (23.9)	37 (40.7)	14 (25.0)	26 (21.5)	
STH prevention							
No idea	14 (24.6)	22 (47.8)	30 (42.3)	28 (30.8)	21 (37.5)	43 (35.5)	0.040
Incorrect answers	11 (19.3)	7 (15.2)	10 (14.1)	21 (23.1)	12 (21.4)	38 (31.4)	
At least one correct answer	32 (56.1)	17 (37.0)	31 (43.7)	42 (46.2)	23 (41.1)	40 (33.1)	
STH signs and symptoms							
No idea	22 (38.6)	21 (45.7)	37 (52.1)	46 (50.5)	29 (51.8)	52 (43.0)	0.720
Incorrect answers	5 (8.8)	5 (10.9)	8 (11.3)	10 (11.0)	8 (14.3)	12 (9.9)	
At least one correct answer	30 (52.6)	20 (43.5)	26 (36.6)	35 (38.5)	19 (33.9)	57 (47.1

### Socio-demographic characteristics of participants who took part in FGDs and in-depth interviews

We conducted 3 FGDs among CCGs and 8 FGDs among caregivers on KAP in relation to schistosomiasis and STH. A total of 62 CCGs, preschool teachers and traditional authorities responded to schistosomiasis and STH KAP interviews. There was a single male respondent among CCGs and a single female respondent among traditional authorities. All preschool teachers were females and the youngest respondent in in-depth interviews was 35 years old. More than two-thirds of CCGs were born in the study area.

### Schistosomiasis awareness, knowledge of signs, symptoms, transmission, life cycle and prevention among caregivers

**Fig. 2:**
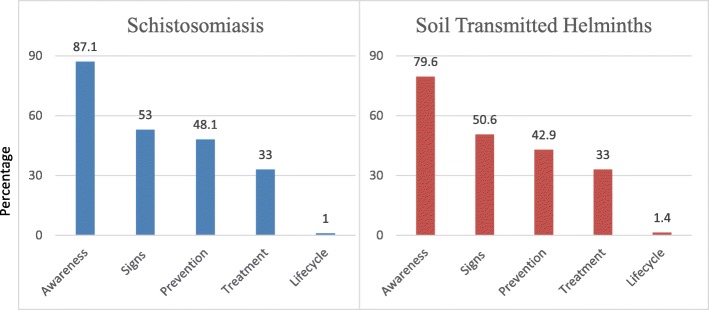
Schistosomiasis and STH awareness, knowledge of signs, symptoms, transmission, life cycle and prevention among caregivers

Most study participants (87.1%) acknowledged having previously heard of schistosomiasis (Fig. 2). Out of these, almost two-thirds knew signs and symptoms (69.1%) and prevention measures (61.6%) of schistosomiasis while half (48.3%) and only 5% knew about treatment and control and life cycle respectively. These notable knowledge gaps arose from study participants who declared not having any information on schistosomiasis ranging from 30.9% on signs and symptoms and 95.1% on the life cycle. Consequently, only about half of the sub-sample that had heard of schistosomiasis could accurately name a specific symptom (53%), knew about prevention (48.1%) while 33% had knowledge on treatment and control and 1% knew could describe the life cycle. Overall, the proportion of sampled participants with knowledge of schistosomiasis ranged from 0.23% on the life cycle to 42.15% on signs and symptoms.

### STH awareness, knowledge of signs, symptoms, transmission, life cycle and prevention among caregivers

**Fig. 3:**
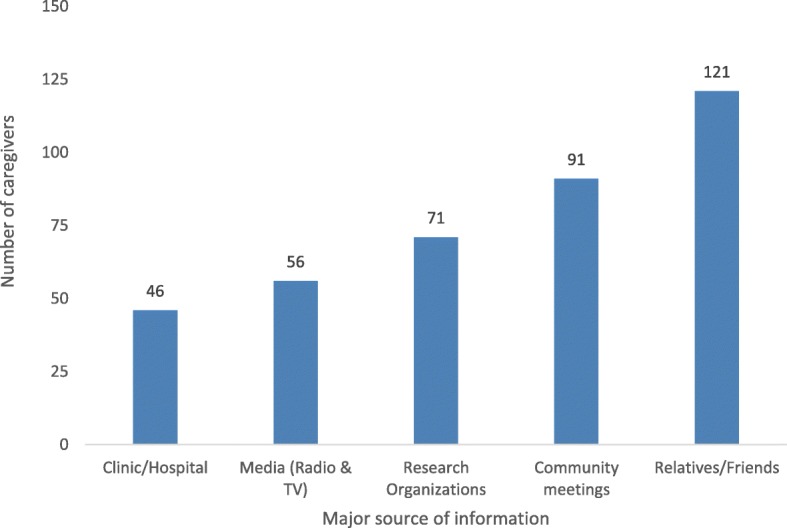
Caregiver’s major source of information on schistosomiasis and STH

Figure 3 presents overall findings on knowledge of soil-transmitted helminths in the Ingwavuma area of uMkhanyakude. Although 79.6% of participants had heard about STH, a large proportion did not have detailed knowledge. Almost two thirds (62.2%) of participants knew some signs and symptoms of STH. However, only 2.3% of participants confirmed knowledge of STH life cycle. Effective knowledge was poor, lower proportions of the sample correctly listed at least a single STH sign/symptom (50.6%), cause (32.4%), life cycle (1.4%) or prevention measure (42.9%).

### Caregivers’ knowledge of schistosomiasis and STH signs, symptoms, life cycle, transmission and prevention

Results show significant misconceptions on signs and symptoms, transmission, life cycle and prevention of schistosomiasis (Table 2). Participants related sexual practices and symptoms of sexually transmitted diseases with schistosomiasis citing genital sores as being symptomatic of schistosomiasis, unprotected intercourse as a transmission mode and protected intercourse as a preventive measure. Other notable misconceptions included transmission of schistosomiasis through feeding practices and including the soil as part of the schistosomiasis life cycle. Misconceptions were also widespread on signs and symptoms, causes, life cycle and prevention of STH. The misconceptions included teeth grinding, dietary habits and treatment as signs, transmission ways and preventive methods respectively.

**Table 2:** Association between caregiver’s knowledge of schistosomiasis and STH and attitudes towards these infections

Caregivers’ attitudes towards schistosomiasis	Caregivers’ knowledge about schistosomiasis prevention and control			
	No idea	Incorrect answers	At least one correct	*X* ^*2*^ *p-value*
Schistosomiasis is a minor infection that may clear overtime without treatment				
Strongly agree	97 (51.3)	30 (15.9)	62 (32.8)	0.001
Somewhat agree	43 (50.0)	10 (11.6)	33 (38.4)	
Somewhat disagree	25 (36.2)	9 (13.0)	35 (50.7)	
Strongly disagree	27 (27.6)	10 (10.2)	61 (62.2)	
Whether schistosomiasis treatment results in severe, long-term side effects				
Strongly agree	107 (46.9)	29 (12.7)	92 (40.4)	0.572
Somewhat agree	31 (39.7)	13 (16.7)	34 (43.6)	
Somewhat disagree	24 (46.2)	5 (9.6)	23 (44.2)	
Strongly disagree	30 (35.7)	12 (14.3)	42 (50.0)	
Schistosomiasis treatment is costly				
Strongly agree	68 (47.9)	16 (11.3)	58 (40.8)	0.015
Somewhat agree	36 (47.4)	16 (21.1)	24 (31.6)	
Somewhat disagree	43 (46.7)	13 (14.1)	36 (39.1)	
Strongly disagree	45 (34.1)	14 (10.6)	73 (55.3)	
Caregivers’ attitudes towards STH	Caregivers’ knowledge of STH prevention and control			
STH are minor infections that cause no harm to the body				
Strongly agree	14 (24.1)	16 (27.6)	28 (48.3)	0.018
Somewhat agree	27 (48.2)	12 (21.4)	17 (30.4)	
Somewhat disagree	29 (44.6)	18 (27.7)	18 (27.7)	
Strongly disagree	88 (33.5)	53 (20.2)	122 (46.4)	
STH only affects adults and not children				
Strongly agree	32 (35.2)	21 (23.1)	38 (41.8)	0.203
Somewhat agree	36 (45.0)	18 (22.5)	26 (32.5)	
Somewhat disagree	31 (39.2)	20 (25.3)	28 (35.4)	
Strongly disagree	59 (30.7)	40 (20.8)	93 (48.4)	
STH treatment is costly				
Strongly agree	37 (43.0)	22 (25.6)	27 (31.4)	0.021
Somewhat agree	38 (45.2)	20 (23.8)	26 (31.0)	
Somewhat disagree	25 (29.8)	21 (25.0)	38 (45.2)	
Strongly disagree	58 (30.9)	36 (19.1)	94 (50.0)	

### Caregivers’ knowledge of schistosomiasis and STH and their main source of information

Out of 385 caregivers who acknowledged to have heard about schistosomiasis and STH, friends/relatives 121(31.4%) and community meetings 91(23.6%) were the most reported as major sources of information while clinics/hospitals were the least reported 46(11.9) source (see Fig. 3).

Caregivers’ knowledge of schistosomiasis and STH and their main source of information Table 3 shows that caregiver’s knowledge on the transmission was associated with the source of information (*p*-value: 0.001) and most participants with correct responses received the information in community meetings. The caregivers’ knowledge of schistosomiasis prevention, signs and symptoms were also associated with the main source of information (p-value: 0.001). Those knowledgeable received the information from community meetings and relatives-friends. For STH, only the knowledge on prevention was associated with the source of information and most of the caregivers were also informed through community meetings.

**Table 3:** Bivariate analysis of STH and Schistosomiasis practices and knowledge levels among caregivers, (*n* = 442)

Schistosomiasis								Soil transmitted helminths					
	Response	Practices		X^*2*^ *p*-value	Knowledge		X^*2*^ *p*-value	Practices		X^*2*^ *p*-value	Knowledge		X^*2*^ *p-*value
		Poor: n (%)			Good: n (%)			Poor: n (%)			Good: n (%)		
Gender	Male	6 (1.4)	22 (4.98)	0.113	26 (5.9)	2 (0.5)	0.222	9 (2.0)	19 (4.2)	0.464	21 (4.8)	7 (1.6)	0.444
	Female	150 (33.9)	264 (59.7)		349 (79.0)	45 (10.2)		107 (24.2)	307 (69.5)		335 (75.8)	79 (17.9)	
Age	15–34	91 (20.6)	188 (42.5)	0.123	238 (53.4)	41 (9.3)	0.722	63 (14.3)	216 (48.9)	0.022	226 (51.1)	53 (12.0)	0.749
	35+	65 (14.7)	98 (22.2)		137 (31.0)	26 (5.9)		53 (12.0)	119 (26.9)		130 (29.4)	33 (7.5)	
Marital status	Married	12 (2.7)	30 (6.8)	0.546	36 (8.1)	6 (1.2)	0.397	13 (2.9)	29 (6.6)	0.708	30 (6.8)	12 (2.7)	0.130
	Widowed	4 (0.9)	10 (2.26)		13 (2.9)	1 (0.2)		3 (0.7)	11 (2.5)		14 (3.2)	0 (0)	
	Single – never married	130 (29.4)	234 (52.9)		305 (69.0)	59 (13.3)		96 (21.7)	268 (60.6)		294 (66.5)	70 (15.8)	
	Divorced	10 (2.3)	12 (2.7)		21 (4.8)	1 (0.2)		4 (0.9)	18 (4.1)		18 (4.1)	4 (0.9)	
Formal education level	None	24 (5.4)	41 (9.3)	0.149	54 (12.2)	11 (2.5)	0.009	20 (4.5)	45 (10.2)	0.838	55 (12.4)	11 (2.5)	0.826
	Primary	32 (7.2)	71 (16.1)		96 (21.7)	7 (1.6)		27 (6.1)	76 (17.2)		82 (18.6)	21 (4.8)	
	Secondary	98 (22.2)	160 (36.2)		209 (47.3)	49 (11.1)		65 (14.7)	193 (43.7)		206 (46.6)	52 (11.8)	
	Post-secondary	2 (0.5)	14 (3.2)		16 (3.6)	0 (0)		4 (0.9)	12 (2.7)		14 (3.2)	2 (0.5)	
Main source of family Income	Grant	136 (30.8)	242 (54.8)	0.731	322 (72.9)	56 (12.7)	0.632	92 (20.8)	286 (64.7)	0.070	306 (69.2)	72 (16.3)	0.357
	Vending	17 (3.8)	36 (8.1)		43 (9.7)	10 (2.26)		19 (4.2)	34 (7.7)		43 (9.7)	10 (2.3)	
	Wage/salary	3 (0.7)	8 (1.8)		10 (2.26)	1 (0.2)		5 (1.1)	6 (1.4)		7 (1.6)	14 (3.2)	
Source of Information	None	21 (4.8)	65 (14.7)	0.003	81 (18.3)	5 (1.1)	0.002	19 (4.2)	38 (8.6)	0.472	56 (12.7)	1 (0.2)	0.013
	Community meetings	21 (4.8)	46 (10.4)		55 (12.4)	12 (2.7)		20 (4.5)	71 (16.1)		34 (7.7)	12 (2.7)	
	Research organizations	14 (3.2)	47 (10.6)		47 (10.6)	14 (3.17)		20 (4.5)	51 (11.5)		57 (12.9)	14 ()	
	Clinic/hospital	10 (2.3)	18 (4.1)		18 (4.1)	10 (2.26)		8 (1.8)	38 (8.6)		69 (15.6)	22 ()	
	Media (Radio/TV)	14 (3.2)	13 (2.9)		23 (5.2)	4 (0.9)		15 (3.4)	41 (9.3)		43 (9.7)	13 ()	
	Relatives and friends	76 (17.2)	97 (21.9)		151 (34.2)	22 (5.0)		34 (7.7)	87 (19.7)		97 (21.9)	24 ()	

### Knowledge, attitudes, practices and control of schistosomiasis and STH

Five themes emerged from the data: (i) preferred sources of information, (ii) knowledge and misconception, (iii) attitudes (iv) water and sanitary practices (v) control and (vi) health-seeking behaviour (see Table 4). Most of the study participants reported to have inadequate knowledge, attitudes on modes of transmission, life cycle, prevention and control “*As for me, I don’t have good information about these diseases, but I remember growing up, our elders used to warn us against jumping over fire and even eating cold food****”*** ; Male respondent – FGD**.** Noted misconceptions were related to the schistosomiasis and STH transmission, signs and symptoms, and prevention. FGD participants mentioned that schistosomiasis may be caused by sexual intercourse, jumping over fire, and drinking dirty water.

**Table 4:** Binary logistic regression analysis of STH and Schistosomiasis practices and knowledge levels among caregivers in Ingwavuma

Disease	Variable	Categories	Practices	Knowledge Level	
			COR (95% CI)	COR (95% CI)	AOR (95% CI)
STH	Formal education level	No formal schooling	–	1	1
		Primary	–	0.935 (0.456–1.918)	1.010 (0.481–2.122)
		Secondary and above	–	0.335 (0.146–0.766)^a^	0.344 (0.148–0.798)^a^
	Source of Information on STH	None	1	1	1
		Community meetings	2.425 (1.363–4.316)^a^	0.424 (0.155–1.161)	0.447 (0.162–1.233)
		Research organizations	1.716 (0.945–3.118)	1.498 (0.695–3.228)	1.632 (0.749–3.555)
		Clinic/hospital	2.630 (1.349–5.130)^a^	2.044 (0.970–4.310)	2.109 (0.993–4.482)
		Media (Radio/TV)	1.410 (0.615–3.232)	3.813 (1.561–9.314)^a^	3.744 (1.518–9.232)^a^
		Relatives and friends	0.728 (0.323–1.639)	1.194 (0.377–3.778)	1.453 (0.450–4.698)
Schistosomiasis	Age group	15–34	1	–	–
		35–70	1.652 (1.073–2.543)^a^	–	–
	Source of Information on STH	None		1	–
		Community meetings		0.072 (0.010–0.548)^a^	–
		Research organizations		1.426 (0.644–3.160)	–
		Clinic/hospital		0.993 (0.476–2.072)	–
		Media (Radio/TV)		1.289 (0.669–2.483)	–
		Relatives and friends		1.222 (0.569–2.625)	–

^a^Significant at 0.05 level

Most participants, however, were able to mention at least one sign or symptom of schistosomiasis and the most mentioned was *“blood in urine”.* Most participants preferred visiting clinics/hospitals as opposed to traditional healers. However, they mentioned that if they do not get better when using modern medicine, they resort to traditional healers or sometimes use both medications at the same time. Noted misconceptions were mostly related to the schistosomiasis and STH transmission and prevention. FGD participants mentioned that Schistosomiasis may be caused by sexual intercourse, jumping over fire, and drinking dirty water.

### Association between caregiver’s knowledge on schistosomiasis and STH and attitudes towards these infections

Additional file 1: Table S1 shows the association between caregiver’s knowledge and attitudes towards schistosomiasis and STH; Respondents who regarded schistosomiasis and STH as minor infections that could heal without any intervention (*p*-value: 0.001) and assumed that schistosomiasis treatment was costly (*p*-value: 0.001) were more likely to have a negative attitude towards schistosomiasis control and prevention. The same trend was also observed with STH.

### Bivariate analysis of STH and schistosomiasis practices and knowledge levels among caregivers in Ingwavuma

Results from the bivariate analysis showed a significant association between age group and source of family income and practices on both STH and schistosomiasis (see Additional file 2: Table S2 and Additional file 3: Table S3). Formal education attainment and source of information was significantly associated with knowledge of STH while the source of health information was the only significant variable for schistosomiasis knowledge.

### Binary logistic regression of STH and schistosomiasis practices and knowledge levels among caregivers in Ingwavuma

From the multivariate analysis, caregivers who got information through community meetings (COR 2.425, 95% CI: 1.363–4.316) and health institutions (COR 2.630, 95% CI: 1.349–5.130) were more likely to report good STH practices than those reporting not receiving any information (see Table 4). Caregivers with at least secondary education were 66% (COR 0.335, 95% CI: 0.146–0.766) less likely to have high knowledge of STH relative to their counterparts without any formal education. On the other hand, caregivers who accessed health information through the media had higher odds (COR 3.813, 95% CI: 1.561–9.314) of attaining high knowledge levels on STH when compared with caregivers not receiving any information. However, there was no noticeable change in the likelihood of attaining higher knowledge levels on STH for caregivers at least secondary education (AOR 3.744, 95% CI: 1.518–9.232) those accessing information through the media (AOR 0.344, 95% CI: 0.148–0.798) after controlling for other variables. Being aged at least 35 years increased the odds of reporting good practices on schistosomiasis by 65% (COR 1.652, 95% CI: 1.073–2.543) while receiving health information through community meetings (COR 0.072, 95% CI: 0.010–0.548) significantly reduced the odds of having good knowledge on schistosomiasis (Additional file 2: Table S2).

## Discussion

### Major sources of information on schistosomiasis and STH

This study shows a significant relationship between caregiver’s major source of information and the ability to give at least one correct answer. Our study revealed that most people relied on family friends and community meetings for health information. Caregivers that could not give accurate responses on schistosomiasis and STH were those that relied on family and friends for schistosomiasis and STH related information. Our findings are in line with previous studies that found that peers and family members tend to give inaccurate health messages [29, 30] due to limited health-related knowledge [31]. This suggests therefore that, if community members are empowered with accurate information, schistosomiasis and STH control programs can leverage on them as existing dissemination channels, in order to halt the spread of infection through behaviour change strategies.

### Knowledge and practices on schistosomiasis and STHs

This study aimed to understand knowledge, attitudes and practices on schistosomiasis and STH among caregivers in rural KwaZulu-Natal, South Africa. Our study provides invaluable insights into KAP in rural South Africa. There was education intervention among participants prior to the study. The establishment of the level of knowledge and misconception prior to an intervention program was important to design context-specific educational programs on schistosomiasis and STH. Most respondents claimed to be aware of schistosomiasis and STH. however, knowledge levels declined drastically with follow-up questions assessing the accuracy of the self-reported knowledge. Knowledge was highest on signs and symptoms and the least on life cycle for both schistosomiasis and STH. The low knowledge was prevalent across categories of key caregivers’ socio-demographic characteristics. The low level of knowledge despite high awareness levels have been reported widely by KAP studies conducted in Bangladesh [32], Kenya [33], South Africa [34] and Ethiopia [35]. A recent systematic review on KAP of schistosomiasis in SSA reported low levels of comprehensive knowledge despite high levels of self-reported disease awareness ranging from between 75 and 98% [15]. This strongly suggests communities may overestimate their health-related knowledge which may, in turn, hinder the effectiveness of treatment and control programs and has implications for health promotion intervention. For this reason, contextual realities ought to be considered when designing health awareness campaigns particularly in rural areas with low levels of formal education. Risky water and sanitary related practices were observed among most of the participants who reported relying on open water sources due to water scarcity and whose children practice open defecation due to the unavailability of appropriate toilets for PSAC. Previous studies have overemphasized the importance of water, sanitation and hygiene (WASH) in the control of schistosomiasis and STH [36]. Disadvantaged communities, however, are still grappling with the provision of such services due to financial constraints and expertise required for their implementation.

### Attitudes and misconceptions on schistosomiasis and STHs infection

Our findings revealed widespread misconceptions on schistosomiasis and STH among all participants and respondents i.e. community caregivers, traditional leaders, preschool teachers, caregivers in the Ingwavuma community. Most fallacies related to schistosomiasis signs and symptoms, transmission and prevention. For instance, participants in FGDs and in-depth interviews confused schistosomiasis with sexually transmitted infections due to the blood in urine. Our findings are consistent with previous findings from other settings including Tanzania [37], Mozambique [38], and Kenya [39, 40]. On the other hand, the few misconceptions on STH were seemingly mythical. Rural communities are often marked by dominant cultural influences characterized by widespread close-knit structures of influence thus wrong messages may easily lead to widespread misconceptions and risky practices that cascade across generations [41]. The distortion of health seeking behaviour resulting from misconceptions and myths on illness may hinder control efforts in the case of schistosomiasis and STH.

Most male participants in our study underestimated the seriousness of schistosomiasis and STH, rating them as infections that not cause death easily. Earlier studies from Uganda [42], Tanzania [37], Kenya [43] and Nigeria [20] had similar findings where respondents believed schistosomiasis was a natural disease that did not require medical attention. In our study, FGDs with men also revealed that they believed it was the responsibility of women to acquire health information because their cultural and ordained role is to take care of their children and the entire family. Contrary to this belief, earlier systematic review findings reported that women had lower levels of health-related knowledge compared to men [15], however, men tend to have poor health-seeking behaviour compared to women [44]. Such variations in beliefs and practices, however, are not surprising considering that in most rural areas in African societies, human behaviour is mainly governed by strong sociocultural influences and expectations [45]. Such beliefs, however, may become bottlenecks for control interventions. Ultimately, they hinder programs from timely controlling and eliminating Schistosomiasis and STH in such settings.

## Conclusion

The low levels of awareness, poor attitudes and practices observed in this study highlight major deficiencies in current strategies to control schistosomiasis and STH. To improve disease control at the community level, it is important to include community stakeholders in developing and delivering awareness programs. This will further enhance the efficiency of communal engagement and information dissemination channels. Developmental interventions as safe water provision are required to minimize exposure to WASH and livelihood activities predispose the community to infection are also required. Finally, it is important to incorporate strategies that address cultural misconceptions and myths on disease into health awareness activities and control measures.

## Additional files

Additional file 1: **Table S1.** Sociodemographic characteristics of caregivers. Describes the overall sociodemographic characteristics of 442 caregivers of PSAC who were screened for schistosomiasis and STH in uMkhanyakude district. (DOCX 16 kb)

Additional file 2: **Table S2.** Caregivers’ knowledge on the signs and symptoms, life cycle, transmission and prevention of schistosomiasis and STH. Is a frequency table showing caregivers’ knowledge of schistosomiasis and STH signs and symptoms, life cycle, transmission and prevention. (DOCX 16 kb)

Additional file 3: **Table S3.** Knowledge, attitudes, practices and control of schistosomiasis and STH. Shows FGDs and in-depth interview themes, descriptions and verbatim quotes from CCGs and caregivers. (DOCX 18 kb)

## Data Availability

The datasets used and/or analysed during the current study are available from the corresponding author on reasonable request.

## Abbreviations

CCG: Community caregiver
CHW: Community health worker
FGD: Focus Group Discussion
KAP: Knowledge, attitudes and practices
PSAC: Preschool age children
SAC: School-aged children
STH: Soil-transmitted helminths
WASH: Water Sanitation and Hygiene
WHO: World Health Organization
