# Supplementary material for: Knowledge, attitudes and practices on schistosomiasis and soil-transmitted helminths among caregivers in Ingwavuma area in uMkhanyakude district, South Africa
Source: BMC Infect Dis. 2019 Aug 22;19:734. doi: 10.1186/s12879-019-4253-3 (PMC6704662; doi:10.1186/s12879-019-4253-3)
Supplement: Supplementary file 1 — Table S1. Sociodemographic characteristics of caregivers. Describes the overall sociodemographic characteristics of 442 caregivers of PSAC who were screened for schistosomiasis and STH in uMkhanyakude district. (DOCX 16 kb) [file 12879_2019_4253_MOESM1_ESM.docx]

**Additional file 1: Table S1** Sociodemographic characteristics of caregivers (n=442)

| **Variables** | **Character** | **Frequency** | **(%)** |
| --- | --- | --- | --- |
| Age in years of caregivers | 15-34 | 105 | 23.8 |
|  | 25-34 | 174 | 39.4 |
|  | 35-44 | 85 | 19.2 |
|  | 45-54 | 44 | 10.0 |
|  | 55-70 | 34 | 7.7 |
| Gender of caregivers | Male | 28 | 6.3 |
|  | Female | 414 | 93.7 |
| Marital status of caregivers | Single-never married | 364 | 82.4 |
|  | Divorced | 22 | 5.0 |
|  | Widowed | 14 | 3.2 |
|  | Married | 42 | 9.5 |
| Education level of caregivers | No formal education | 65 | 14.7 |
|  | Primary school | 103 | 23.3 |
|  | Secondary school | 258 | 58.4 |
|  | College level | 16 | 3.6 |
| Occupation of caregiver | Not working | 372 | 84.2 |
|  | Self employed | 39 | 8.8 |
|  | Employed | 31 | 7.0 |
| Major source of family income | Grants | 379 | 85.7 |
|  | Street vending | 56 | 12.7 |
|  | Earning monthly salary | 7 | 1.6 |
