# Supplementary material for: Knowledge, attitudes and practices on schistosomiasis and soil-transmitted helminths among caregivers in Ingwavuma area in uMkhanyakude district, South Africa
Source: BMC Infect Dis. 2019 Aug 22;19:734. doi: 10.1186/s12879-019-4253-3 (PMC6704662; doi:10.1186/s12879-019-4253-3)
Supplement: Supplementary file 2 — Table S2. Caregivers’ knowledge on the signs and symptoms, life cycle, transmission and prevention of schistosomiasis and STH. Is a frequency table showing caregivers’ knowledge of schistosomiasis and STH signs and symptoms, life cycle, transmission and prevention. (DOCX 16 kb) [file 12879_2019_4253_MOESM2_ESM.docx]

**Additional file 2: Table S2** Caregivers’ knowledge on the signs and symptoms, life cycle, transmission and prevention of schistosomiasis and STH (n=442)

| **Questions** | **Responses** | **Frequency** | **%** |
| --- | --- | --- | --- |
| Schistosomiasis signs and symptoms | No knowledge | 119 | 30.9 |
|  | Blood in urine | 180 | 46.8 |
|  | Loss of weight | 31 | 8.1 |
|  | Genital sores | 31 | 8.1 |
|  | Pain when urinating | 17 | 4.4 |
|  | Abdominal pain | 7 | 1.8 |
| Schistosomiasis life cycle | No knowledge | 366 | 95.1 |
|  | The parasite grows in soil | 15 | 3.9 |
|  | The parasite requires a snail to grow | 4 | 1 |
| Schistosomiasis transmission | No knowledge | 197 | 51.2 |
|  | Swimming/bathing in infected water | 127 | 33 |
|  | Drinking dirty water | 23 | 6 |
|  | Unprotected sex | 15 | 3.9 |
|  | Jumping over fire | 13 | 3.4 |
|  | Eating too much | 10 | 2.6 |
| Schistosomiasis prevention | No knowledge | 148 | 38.4 |
|  | Avoid washing in infected water | 131 | 34 |
|  | Avoid swimming/bathing in infected water | 54 | 14 |
|  | Avoid drinking dirty water | 33 | 8.6 |
|  | Use protection during sexual intercourse | 15 | 3.9 |
|  | Taking treatment to avoid infection | 4 | 1 |
| STH signs and symptoms | No knowledge | 135 | 38.4 |
|  | Abdominal distention | 58 | 16.5 |
|  | Skin rash | 50 | 14.2 |
|  | Abdominal pain | 48 | 13.6 |
|  | Teeth grinding | 39 | 11.1 |
|  | Diarrhoea | 22 | 6.3 |
| STH life cycle | No knowledge | 344 | 97.7 |
|  | It lives in the soil | 5 | 1.4 |
|  | It lives in steady water | 3 | 0.9 |
| STH transmission | No knowledge | 189 | 57.7 |
|  | Eating contaminated food | 80 | 22.7 |
|  | Eating soil | 34 | 9.7 |
|  | Giving children hard food | 28 | 8 |
|  | Hunger | 14 | 4 |
|  | Drinking too much milk/sour milk | 7 | 2 |
| STH prevention | No knowledge | 132 | 37.5 |
|  | Washing hands before eating | 77 | 21.9 |
|  | Treatment may prevent infection | 69 | 19.6 |
|  | Avoid playing in soil | 43 | 12.2 |
|  | Avoid drinking dirty water | 31 | 8.8 |
