# Supplementary material for: Knowledge, attitudes and practices on schistosomiasis and soil-transmitted helminths among caregivers in Ingwavuma area in uMkhanyakude district, South Africa
Source: BMC Infect Dis. 2019 Aug 22;19:734. doi: 10.1186/s12879-019-4253-3 (PMC6704662; doi:10.1186/s12879-019-4253-3)
Supplement: Supplementary file 3 — Table S3. Knowledge, attitudes, practices and control of schistosomiasis and STH. Shows FGDs and in-depth interview themes, descriptions and verbatim quotes from CCGs and caregivers. (DOCX 18 kb) [file 12879_2019_4253_MOESM3_ESM.docx]

**Additional file 3: Table S3** Knowledge, attitudes, practices and control of schistosomiasis and STH

| **Themes and descriptions** | **Disease** | **Verbatim quotes** |
| --- | --- | --- |
| **Preferred sources of information**: Most respondents preferred being taught during community meetings and by using the house to house strategy | **Schistosomiasis** | *“The best would be community meetings because everyone comes but they don’t teach us these things, as for me I got to know from my relative”* **Female respondent - FGD**  *“Woooo as for those written paper messages, I don’t think they would work here among us old people, some of us cannot read and we cannot even see things clearly anymore”* **Male traditional authority- In-depth interview** |
|  | STH | *I think CCGs would be the best because they enter house by house educating us about many things, but I have not heard them talking about bilharzia and soil-transmitted helminths”* **Female preschool teacher-In-depth Interview** |
| **Knowledge and Awareness**- Most respondents demonstrated a low level of knowledge on the prevention, life cycle and transmission of both STH and schistosomiasis. CCGs had better knowledge compared to preschool teachers and traditional authorities | Schistosomiasis | “*Most community members are familiar with the disease name Isichenene (bilharzia) but we don’t really know a lot about it*” **Female respondent – FGD**  *“Personally, I am not sure about these diseases because they are not famous in our community, but I know that one can become infected through swimming in a river and by drinking dirty water”* **Female CCG - In-depth interview**  *“As for me, I don’t have good information about these diseases, but I remember growing up, our elders used to warn us against jumping over fire and even eating from a pot”* **Male respondent – FGD** |
|  | STH | *“What I can remember is that stomach worms may sometimes be caused by eating cold food”* **Female CCG-In-depth interview.** |
| **Attitudes and misconceptions-**There were notable misconceptions and negative attitudes. Some respondents confused schistosomiasis with STIs due to the blood in urine whilst some underestimated the seriousness of both infections. | Schistosomiasis | *“I must say that I don’t think this disease is taken seriously in our community because since I was born, I have never heard of anyone who died of bilharzia, but I know people who died from HIV, TB, stroke and diabetes”* **Female CCG- In-depth interview** |
|  | STH | *“Women should know better these things better because they are the ones who take care of children, these are childhood diseases”* **Male respondent – FGD** |
| **Water and sanitary practices** – Most respondents were aware of the dangers of drinking unclean water however almost all of them stated that they had no better alternative and that it is not always possible to treat water meant for drinking | Schistosomiasis | *“Here we mainly depend on river water, during the dry season we dig trenches and we access to water, this water is dangerous, and we know that but there is nothing we can do because honestly, water shortage is a real struggle in our community”*- **Male respondent – FGD**  *The biggest problem is that even the community taps that government gave to us have been lacking water for a very long time now, some have taps in their homes, but water can’t come out, we all end up relying on water from dams and rivers where we can get cholera and bilharzia***- female CCG- In-depth interview** |
|  | STH | *We all have toilets, the government built us toilets but most of us old people still prefer using the forest because it is not easy to adjust to the modern way of doing things, really it is not easy*- **Male respondent – FGD**  *“Another problem is that our young children cannot use these pit toilets because they are very dangerous and because they are young they cannot defecate away from home, they will find a spot around the boundaries where it is safe and our chickens always feed on their faeces so in that way we get the soil-transmitted helminths***” Female respondent – FGD** |
| **Control**- People knew that these diseases are treatable but thought the treatment was either too expensive or resulted in severe side effects. Some respondents believed that treatment could confer temporal/long-term immunity towards the disease. | Schistosomiasis | *“Bilharzia can be treated through tablets from the clinic, the problem is that they say these tablets make you suffer from stomach ache and headache too much before you can be cured”* **Female respondent- FGD**  “*Yah some even vomit and become sick, but the good thing is that once you take the treatment, you can be protected from bilharzia for a long time”* **Male respondent – FGD** |
|  | STH | “*Children in primary schools receive the tablets to prevent and treat STH for free, nurses give them in schools every year, but younger children and adults can only be treated at the clinic or they can buy from the pharmacy”* **Female preschool teacher- In-depth Interview** |
| **Health seeking behaviour**- Most respondents relied on clinics for medical care however they stated that delayed treatment due is the main challenge because people may not visit the clinics early due to lack of knowledge. Other reasons were lack of money and lack of time. | Schistosomiasis | *“Most people here use the clinics for health care but sometimes we delay out of ignorance, we only go to the clinic when the condition becomes serious”* **Male traditional leader- In-depth interview**  *“People here use traditional medicine after failing to recover from their illness when using medicine from the clinic”* **Female respondent- FGD** |
|  | STH | *“Sometimes it is difficult to visit the clinic because of fear, when I suffered from bilharzia some years back I was afraid of telling people that my urine had blood, I thought it was a sexually transmitted disease, it took me a very long time to visit the clinic”* **Male respondent – FGD** |
